# Bullying Victimization among Mexican Adolescents: Psychosocial Differences from an Ecological Approach

**DOI:** 10.3390/ijerph17134831

**Published:** 2020-07-04

**Authors:** Silvana Mabel Nuñez-Fadda, Remberto Castro-Castañeda, Esperanza Vargas-Jiménez, Gonzalo Musitu-Ochoa, Juan Evaristo Callejas-Jerónimo

**Affiliations:** 1 Department of Psychology, Coast University Center, University of Guadalajara, Puerto Vallarta 48280, Mexico; reembert@hotmail.com (R.C.-C.); esperanzavgas@hotmail.com (E.V.-J.); 2 Department of Education and Social Psychology, Pablo de Olavide University, 41013 Seville, Spain; gmusoch@upo.es (G.M.-O.); jevaristocallejas@gmail.com (J.E.C.-J.)

**Keywords:** bullying victimization, parents–adolescent communication, psychological distress, attitude toward authority, community social support, gender

## Abstract

This transversal study over a random representative sample of 1687 Mexican students attending public and private secondary schools (54% girls, 12–17 years old, *M* = 13.65. *DT* = 1.14) aimed to analyze psychosocial differences between victims and non-victims of bullying from the bioecological model. It included individual variables (ontosystem), familiar, community, and scholar factors (microsystem), and gender (macrosystem) to perform a multivariate discriminant analysis and a logistic regression analysis. The discriminant analysis found that psychological distress, offensive communication with mother and father, and a positive attitude toward social norms transgression characterized the high victimization cluster. For the non-victims, the discriminant variables were community implication, positive attitude toward institutional authority, and open communication with the mother. These variables allowed for correctly predicting membership in 76% of the cases. Logistic regression analysis found that psychological distress, offensive communication with the father, and being a boy increased the probability of high victimization, while a positive attitude toward authority, open communication with the mother, and being a girl decrease this probability. These results highlight the importance of open and offensive communication between adolescents and their parents on psychological distress, attitude toward authority, community implication, and bullying victimization.

## 1. Introduction

The research about bullying victimization shows that the most prejudicial consequences in development and health impacts on the victims [1,2]. These adverse effects fall into the leading mental health problems worldwide [3], including internalizing problems, such as depression, anxiety, and suicidal behavior [4,5,6,7], and externalizing ones, such as violent and delinquent behavior, substance use disorders, and sexual risk behavior [4,7,8,9].

Bullying is defined as intentional damage inflicted for one or more students on others, without previous provocation, that is persistent in time and occurs within an unbalance of power [10]. The studies about the effects of bullying on victims agree that the unbalance of power is a significant cause of the damage [11,12]. This difference of power is best determined for the victims’ subjective perception that it is impossible for them to get out of the situation or to defend themselves [13]. Some of the characteristics that can facilitate becoming a target of bullying are physical disabilities, mental health problems, and being part of a minority by ethnicity, religious beliefs, or different sexual orientation [2,14]. Empirical research studies indicate that victims of bullying present low self-esteem and self-concept, academic problems, and poor social skills, which can hinder the relationships with their classmates and teachers [15,16]. These characteristics could be pre-existing trends that facilitate social isolation and rejection by pairs but also are a consequence of bullying, increasing the power unbalance between victims and perpetrators [17].

Although bullying occurs mainly in the school context, their links with other developmental environments, especially family and community, are well established by empiric research [2,14]. Ecological models are especially useful in the study of bullying because they offer an organized frame to examine reciprocal interactions between multiple variables at different levels. The bioecological model of human development [18] considers an individual level (ontosystem), and their interactions with the proximal levels (microsystem) constituted mainly by family, school, and community. They interact with more distal contexts, for example, with workplaces of the parents (exosystem), within socio-cultural frames that influence all the others, such as gender (macrosystem), in dynamic evolutions across time (chronosystem). Interactions between different microsystems (such as family and school) constitute the mesosystem.

At the family microsystem level, open and warm communication between parents and adolescents, which express and facilitate a positive family climate, promotes in the adolescents the perception of belonging, acceptance, and agency, facilitating socialization, and protecting them directly and indirectly from involvement in bullying at school [19,20]. Difficulties in parents–adolescent communication, with high levels of offensive communication and verbal harshness, are often associated with the use of physical and psychological punishment from parents. They determine an adverse family climate, hampering development in general [21], particularly emotion socialization processes, by favoring emotion dysregulation and aggressive behavior [22].

Emotion dysregulation in adolescence is strongly related to psychological distress [23], defined as psychological suffering characterized by symptoms of anxiety and depression, with a range of dysfunction from mild to severe at cognitive, emotional, and behavioral levels of functioning [24,25]. There is evidence that supports the correlation between psychological distress and victimization [26,27], but also with bullying perpetration. The possibility of a common path between perpetrators and victims seems to point out toward harsh parental practices, presents in the authoritarian and negligent parental styles [26,27], and family violence [22,28,29].

The experience of predictably benevolent parents determines in the children a secure attachment and the development of “epistemic trust” [29,30,31] that facilitates the transmission from parents to children of relevant information for the socialization process, as social rules. Confidence in the ability of adult caretakers of notice and sensibly respond to their child’s needs is later generalized to adults and authority figures at other socialization environments, such as the school and community, determining a positive attitude toward authority [32]. The positive attitude toward institutional authority (PAIA) is a protective factor toward involvement in bullying, either as a perpetrator [33,34] or as a victim [35,36]. On the contrary, a positive attitude toward social norms transgression (PASNT) is associated with involvement in violent behavior at school [37,38] and also with victimization [39].

Open communication with their parents also favors adolescents’ social skills and the inclusion in their neighborhoods, showing a positive correlation with the perception of community implication, participation, and informal systems support [40]. Adolescents that feel integrated and participants at their communities report better scores in global and social self-concept and life satisfaction, and lower scores in loneliness [15]. Community implication, an affective evaluative dimension that denotes the feeling of bonding and belonging with the neighborhood, is particularly crucial in this respect [15,41]. The beneficial effects of the community social support on social skills, self-concept, and wellbeing can explain their protective effect over school victimization [42].

Gender is a socio-cultural construction of differences between sexes that generate inequities of rights and obligations. Because it is a macro systemic variable, gender affects all the other factors in the study. For example, boys are more involved in bullying than girls, as perpetrators and victims [2,38,43], especially concerning physical and verbal victimization. While girls obtain higher means in PAIA, boys report higher means of PASNT [38]. Girls report higher scores in psychological distress than boys [36,44]. Gender affects the relationship with parents, teachers, and peers through gender stereotypes that define the ideals of masculinity and femininity, assigning the roles of mothers and fathers, daughters and sons, and molding the relationships between them [36,45,46].

### The Present Study

Although several studies have established the relations between these variables and school bullying, fewer have examined their relations with bullying victimization among Latin American adolescents from an ecological frame. In consequence, the aim of this study is analyzing the psychosocial variables that differentiate the adolescent victims and no victims of bullying (school microsystem), including psychological distress (ontosystem), attitude toward authority (school microsystem), parents–adolescent communication (family microsystem), perceived social support (community microsystem), and gender (macrosystem). The hypotheses proposed are: psychological distress, offensive communication with parents, and PASNT will be associated with membership to the highly victimized group (H1). Open communication with parents, PAIA, and perceived social support (community implication, community participation, and informal systems support) would be associated with no victimized group (H2). Psychological distress, offensive communication with parents, PASNT, and being a boy will increase the likelihood of victimization, while open communication with parents, PAIA, perceived social support, and being a girl will reduce the likelihood of victimization (H3). These variables will show significant differences by gender (H4).

## 2. Materials and Methods

It is a transversal, not experimental, observational study.

### 2.1. Participants

The size of the representative sample for the students of secondary schools of Puerto Vallarta, Mexico, (*n* = 14,759), was calculated in 1685 adolescents (error ±2.5%, confidence level 95%, 0.5 variance). The participants (54% girls) were between 12–17 years old (*M* = 13.65. *DT* = 1.14). Students attended the first (*n* = 542), second (*n* = 573) and third (*n* = 568) years of secondary school. Four students did not indicate their level but completed the questionnaire. Educative centers (10 publics and three privates) were selected by bi-staged conglomerate sampling [47] from an official list provided by the regional educational authority.

### 2.2. Instruments

#### 2.2.1. Parents–Adolescent Communication Scale, Spanish Adaptation 

The original [48] scale, and the spanish version [49] are composed of two sub-scales, for communication with the mother and the father, respectively. It has 20 Likert-type items ranging from 1 (never) to 5 (always), grouped in three dimensions: Open communication (i.e., “I can express my true feelings to her/him”), offensive communication (i.e., “She/he says things that hurt me”) and avoidant communication (i.e., “I do not dare to ask her/him for what I wish or want”). A confirmatory factorial analysis (CFA) with the maximum likelihood method verified the three-dimensional structure of the two subscale, showing a good and acceptable adjustment to the data (communication with mother SB χ^2^ = 474.4821, gl = 143, *p* < 0.001, CFI = 0.950, RMSEA = 0.046 (0.041, 0.050), and communication with Father SB χ^2^ = 548.4464, gl = 148, *p* < 0.001, CFI = 0.952, RMSEA = 0.049 (0.045, 0.054)). In this study, open and offensive communication subscales were used, with Cronbach’s alpha of 0.93 for open communication with mother (OPCM), 0.93 for open communication with father (OPCF), 0.73 for offensive communication with mother (OFCM), and 0.72 for offensive communication with father (OFCF).

#### 2.2.2. Adolescent Attitudes towards Institutional Authority Scale (AAI-A) 

The scale [50] is composed of nine Likert-type items with answers from 1 (totally disagree) to 5 (totally agree). It has two subscales: a positive attitude toward institutional authority (PAIA) (i.e., “Police are for making society better for all”) and a positive attitude toward social norms transgression (PASNT) (i.e., “If you do not like a school rule, it is best not to follow it”). A confirmatory factorial analysis (CFA) with the maximum likelihood method verified the two-dimensional structure of the scale, showing a good adjustment to the data (SB χ^2^ = 92.4382, gl = 25, *p* < 0.001, CFI = 0.972, RMSEA = 0.040 (0.032, 0.049)). In this study, the Cronbach’s alphas were 0.75 for PAIA and 0.74 for PASNT.

#### 2.2.3. School Victimization Scale 

The scale [51] is composed by 22 Likert-type items, with responses from 1 (never) to 4 (always) distributed in three factors, relational victimization (i.e., “A schoolmate has told others not to be my friends”), direct physical victimization (i.e., “A schoolmate hit me”), and direct verbal victimization (i.e., “A schoolmate insulted me”). Items 21 and 22 inform about repetition and frequency of victimization in the previous year. A confirmatory factorial analysis with the maximum likelihood method verified the three-dimensional structure of the scale, showing a good adjustment to the data (SB χ^2^ = 293.7139, gl = 142, *p* < 0.001, CFI = 0.952, RMSEA = 0.031 (0.026, 0.036)). In this study, the Cronbach Alpha was 0.92 for relational victimization, 0.72 for direct physical victimization, 0.85 for direct verbal victimization, and of 0.95 for the full scale.

#### 2.2.4. Kessler Psychological Distress Scale K10 

The original scale [52] was adapted to Spanish [53] and validated in Mexican adolescents [36]. It includes ten items with Likert-type answers from 1 (never) to 5 (always) that evaluate anxious and depressive symptoms (i.e., “How often have you felt hopeless?”). A confirmatory factorial analysis with the maximum likelihood method verified the two-dimensional structure of the scale, showing an acceptable adjustment to the data (SB χ2 = 120.9903, gl = 30, *p* < 0.001, CFI = 0.983, RMSEA = 0.043 (0.045, 0.051)). In this study, the Cronbach Alpha was of 0.95.

#### 2.2.5. Community Social Support Scale 

The scale [54] consists of 24 items with Likert-type answers from 1 (totally disagree) to 4 (totally agree). In this study, we included three subscales: community implication (i.e., “In my neighborhood they appreciate me”), community participation (i.e., “I use to participate in the activities organized in my neighborhood”), and informal systems social support (i.e., “In my neighborhood there are people that can help me to solve my problems”). A confirmatory factorial analysis with the maximum likelihood method verified the three-dimensional structure of the scale, showing an acceptable adjustment to the data (SB χ^2^ = 708.7472, gl = 162, *p* < 0.001, CFI = 0.928, RMSEA = 0.043 (0.040, 0.047)). In this study, the Cronbach alpha was 0.68 for community implication, 0.68 for community participation, 0.79 for informal systems social support, and of 0.82 for the full scale.

### 2.3. Procedure

Researchers of the University of Guadalajara (Mexico) and Pablo de Olavide (Spain) designed the study. After approval by the bioethical committee of Coast University Center (University of Guadalajara), and with the support of school authorities, an informative letter and informed consent were sent to parents of the selected students.

Data collection took place during March and April of 2016. The questionnaires were applied by the researchers, at the classrooms of the selected groups, during school hours, scheduled by teachers. The study’s goals were explained to the students, as well as voluntary and anonymous participation. Adolescents authorized by their parents that consent to participate filled out the scales in their usual classrooms within a 60-min period. The study met the ethical values required for research on human beings, following the updated principles of the Helsinki Declaration [55]. The participation rate was 99%.

Missing values were treated by regression imputation method [56] if they were not above 20% of the scale. If such values were higher than 20%, the scale was excluded for the subject. If it were more than two scales excluded by missing or atypical data, the subject was excluded from the study. Univariate atypical data were detected by exploration of standardized scores [57]. In total, eleven subjects were excluded from the study, seven because of missing values and four due to atypical values, giving a final sample of 1687 adolescents.

### 2.4. Data Analysis

The first step was a descriptive statistical analysis. The means and standard deviation of some of the variables studied had differences by gender, confirmed by the *t* Student proof. The Pearson analysis found significant correlations between all the variables in the study. Bi-stage cluster analysis for victimization grouped students in three sets: high victimization, moderate victimization, and no victimization. A discriminant analysis was carried out to determine which of the variables discriminate better between the clusters of high victimization and no victimized adolescents, establishing the saturation cut-off point in 0.30 or above. Finally, hierarchical logistic regression was calculated for the total sample. Statistical analyses were performed with SPSS 25.

## 3. Results

### 3.1. Pearson Correlations and t Student Proof

All the variables included showed significant correlations (Table 1). The *t* Student proof showed significant differences between girls and boys. Boys reported higher scores in direct physical and verbal victimization, OFCF, community implication, and PASNT. The girls scored higher only in psychological distress.

The strongest correlations correspond to Psychological Distress (PD) and relational and verbal victimization. The negative relations between PD and open communication with mother and father, and a positive relation between offensive communication with mother and PD were also important. Another relevant relation was found between OPCM, OPCF an PAIA, and between PAIA and community implication. For PASNT, the most relevant relations were a positive relation with OFCM and OFCF.

### 3.2. Discriminant Analysis

A discriminant analysis was performed to find the linear combination of the variables that best differentiate the scores in the independent variable (School victimization). It obtains significant differences between the high victimization and no victimization clusters for the following discriminant variables: psychological distress, open and offensive communication with mother and father, PAIA and PASNT, community implication, community participation, and informal systems social support. The M de Box test was significant (*F* (55,67201.507) = 99.513; *p* < 0.001), discarding null hypothesis that matrixes of population covariance were equal. Lambda of Wilks coefficient was significant (*X*^2^_(10)_ = 134.050; *p* < 0.001), which rejected the null hypothesis of equality between the clusters in the means of the discriminant variables. The canonic correlation coefficient was η^2^ = 0.33, confirming the validity of the model to discriminate between the two clusters. Table 2 shows the coordinates of the centroid projection of each group over the discriminant function.

These coefficients indicate the number of standard deviation in that the means of each group deviate from the central mean for the entire sample. As this distribution is normalized, the mean equals 1, and the sigma equals 0. In the function obtained, the no victimized adolescents deviate from the center in the opposite direction that the adolescents show high victimization.

The matrix structure with the variables, ordered by degree of canonic correlation (saturation) with the discriminant function is shown in Table 3; the cutoff point is highlighted in green. Psychological distress, offensive communication with mother and father, and PASNT showed saturations in the highly victimized group. Instead, the variables of community implication, PAIA, and OPCM showed saturations in the no victimized group. Although the variables OPCF, informal-systems social support, and community participation are present in the no victimized cluster, their values are below the established cut-off point of 0.30.

Finally, to know the quality of the prediction, the results of group classification are presented in Table 4. The prediction was correct in 75.8% of the students. It correctly predicts the belonging to the no victims group in 76% of the students, and to the high victimized group in 72.6%.

### 3.3. Regression Analysis

The results of the regression analyses (stepwise) to predict bullying victimization are displayed in Table 5. As predictive variables, we used those that reached or surpassed the cutting off point (0.3) at the discriminant analyses. At first, the variables of gender and age were included. The model obtained was significant *F* (2,1553) = 4.255, *p* < 0.05. Being a woman (*β* = −0.074; *p* < 0.001) explained 0.5% of the variance of the victimization (R^2^ = 0.005).

At the second step, psychological distress, PAIA and PASNT variables were included. Being a woman (*β* = −0.134; *p* < 0.001), along with Psychological distress (*β* = 0.359; *p* < 0.001), PAIA (*β* = −0.119; *p* < 0.001), and PASNT (*β* = 0.072; *p* < 0.01), significantly contributed to the prediction model *F* (5,1550) = 67.357; *p* < 0.001, which explained 17.6% of the variance. In a third step, the parents–adolescent communication variables were included: OPCM, OFCM and OFCF. Again, being a woman (*β* = −0.132; *p* < 0.001), psychological distress (*β* = 0.326; *p* < 0.001), and PAIA (*β* = −0.083; *p* < 0.01), along with OPCM (*β* = −0.059; *p* < 0.05), and OFCF (*β* = 0.113; *p* < 0.01), contributed to the predictive model *F* (8,1547) = 46.981, *p* < 0.001, which explained 19.5% of the variance.

Finally, at the fourth step, the community implication variable was included. Once more, being a woman (*β* = −0.135; *p* < 0.001), psychological distress (*β* = 0.322; *p* < 0.001), PAIA (*β* = −0.077; *p* < 0.01), OPCM (*β* = −0.052; *p* < 0.05) and OFCF (*β* = 0.110; *p* < 0.01) contributed to the predictive model *F* (9,1546) = 42.106, *p* < 0.001, which explained 19.7% of the variance of victimization.

## 4. Discussion

This study aimed to analyze, from an ecological approach, the psychosocial variables that allow for differentiating adolescent victims and non-victims of bullying, including psychological distress, parents–adolescent communication with mother and father, PAIA, PASNT, perceived community support, and gender.

The results confirm H1, showing that psychological distress, offensive communication with mother and father, and an attitude favorable to social norms transgression predicted membership to the high victimization group. Concerning H2, it was partially confirmed by the results because only community implication, OPCM, and PAIA predicted membership to the no victims group. Community participation, informal system support, and open communication with the father did not reach the established cut-off value.

The logistic regression analysis found that psychological distress, OFCF and being a boy increased the probability of high victimization, and positive attitude toward authority, open communication with the mother, and being a girl decrease this probability, partially confirming H3, since the other variables included in the study (OPCF, OFCM, PASNT and the community support variables) were not significant. These results and *t* Student proof partially confirm Hypothesis 4 that it will be significant differences by gender for parents and children. Gender differences were significant only regarding psychological distress, physical and verbal victimization, OFCF, community implication, and PASNT. In the other variable included, there were no significant differences by gender.

The frequent association of school victimization, especially in the bully victims, with conditions of disadvantage or low functionality, has led to questioning if the negative consequences on psychosocial adjustment should be attributed to the effect of bullying, or these preexistent conditions instead [2]. Anxiety negatively affects social interactions and often appears associated with low social acceptance and rejection by peers [17]. These adolescents can develop a hypersensitivity toward rejection in social interactions, which leads to very intense emotional reactions, associated with negative cognitive anticipation toward social interactions that, in turn, increase their anxiety [58]. These interactions show the bi-directionality of the correlation between psychological distress and the negatives experiences in the relational environments of victimized adolescents, allowing a better understanding of their lower social acceptance and perceived social support and higher social exclusion by peers [59].

Offensive family communication and an adverse family climate are important risk factors for bullying victimization [19,20,60]. Several trajectories have been proposed to explain this influence. Persistent offensive communication with parents, characterized by verbal harshness, affects emotion socialization, which explain its link with psychological distress, and also impairs the development of epistemic trust, the foundation of a positive attitude toward authority [35]. Psychological distress, which is characterized by anxiety and depression, predisposes adolescents to more disruptive responses, increasing difficulties in conflict resolution and unregulated coping strategies, like rejection and hostility from parents, which in turn predicts an increase in the symptoms [61]. At the school’s relational level, these symptoms can negatively influence social acceptance, cause social isolation, and facilitate peer victimization. These circumstances could also increase psychological distress in an adolescent that face social relationship with fear and distrust. It has been proposed that the attachment problems give place to an epistemic distrust, meaning a distrust toward social communication in general, that hampers social learning and the ability to ask and receive help from the adults [31,51,52,53]. The distrust toward the parents will be extended to other adults and normative systems, explaining the presence of PASNT in the high victimization group. This relevant finding can explain simultaneous victimization and bullying in adolescents, as in the bully victims, and changing role from victim to perpetrator, related with the disappointment with parents and teachers that fail in their function of protection and support [39].

In this sense, the presence of PASNT is a very notable finding in our study, that lead us to think in the possibility that a high number of the adolescents involved in school violence in our study could be aggressive victims, as has been observed in studies from Mexico [43] and other countries [62,63]. It also brings information on the trajectory from victimization and future externalizing problems, and the long-term evolutions toward delinquent behavior and substance abuse, aside from other mental health problems. The association between psychological distress and delinquent behavior has been confirmed in young law offenders [64]. The lack of trust in adults and social norms systems increases adolescents’ vulnerability to being involved in bullying as a perpetrator and as a victim [35,37,38].

In the discriminant analyses, community implication has a prominent place in the no victims group. The presence of this factor suggests that adolescents with better family communication, and hence a better family functioning, has reached in this stage certain degree of autonomy in social functioning that results in the sense of belonging to their neighborhoods, where they perceive themselves as competent and appreciated, and also have more social support resources. Their effects over another positive development indicators, such as social and academic self-concept and life satisfaction [15,65,66], would protect these adolescents, making them less susceptible to bullying victimization, or, facing peers’ violence, could help them to find adequate and effective strategies to cope, preventing its persistence through time. We think that this factor reflects a high level of social and relational competence in these adolescents, rooted in open communication with parents, which expresses acceptance and support and foster the development of epistemic trust toward adults, authorities and social norms.

There are some interesting differences linked to gender, confirming H4. Regarding parents–adolescent communication, open communication with the mother is present in the non-victim’s clusters, while open communication with the father does not reach significance. This difference could reflect cultural gender differences in the role and consequent expectations assigned to mothers and fathers in Latin America and Mexico, following a more traditional family model [67] where mothers are mainly responsible for the emotional care of their children. In consequence, OPCM could be more influential in the positive aspects of development. Some research points out that OPCM has a stronger relation with PAIA for boys than for girls, maybe because it depends more singularly on the quality of the relationship with mothers. Because girls are more sensitive to broader social context demands, this relation is less potent for them [35]. Moreover, boys are expected to be more independent from the social influence, and the rebellion against social norms (and the consequent transgression) could increase their social reputation among peers [16].

Developmental psychology research has found that emotional dysregulation is a common factor for both externalizing and internalizing problems [68]. Our findings could be explained by the link between offensive communication with parents, psychological distress in adolescents (as evidence of the internalizing expression), and the PASNT, a characteristic that will facilitate the appearance of externalizing disorders [27]. Differentiated effects in boys and girls could be explained by cultural gender roles, leading preferentially toward externalizing expression in boys (disruptive behavior linked to emotional dysregulation) and internalizing in girls (anxiety and depression) [69]. In our study, this is confirmed by the higher means of psychological distress for girls, and for PASNT in boys in the *t* Student proof. It is worth noting the higher means for physical and verbal victimization, and for OFCF in the boys, in the same line of several studies [2,38,43] showing that boys suffer more direct victimization than girls, in schools as well as in a family context.

These gender differences help us to understand why the results of the regression analysis indicate that being a girl, combined with OPCM and PAIA, lower the probability of bullying victimization. Our results lead to us to assume that the adolescents that have developed epistemic trust in social communication will also approach their peers and other adults in a confident and emotionally regulated way. This is evident in their perception of community implication since this refers to the emotional dimension of belonging and positive bonding with their environment. In this study, boys have informed a statistically higher mean of community implication than girls. The fact that, in Mexico, boys are less restrained in their outside home activities than girls, making it easier for them to interact with their neighborhoods could explain this finding. This combination of discriminating factors from the non-victimized group allows us to affirm that open communication with parents, especially with the mother, will result in a predominance of trust, adequate socialization, understanding and acceptance of the rules of different microsystems and, as a consequence, of integration into the community. In the school environment, trust in adults and their norms, and social and emotional regulation skills will favor both, social acceptance by peers and the support of teachers, protecting these adolescents from being victims of bullying.

On the contrary, the results allow for noticing how disadvantage factors are grouped in the high victimization cluster (offensive communication with mother and father, a high degree of psychological distress, and a positive attitude towards social norms transgression, all of which involve mistrust in social communication in general). Why is OFCF significant in the regression analysis, while OFCM is not? One possibility is that Mexican mothers, aside from the harshness in communication, also bring warmth and affection to their child, which could modulate the effects of the first [70]. Because traditional masculinity prevents the expression of affection in fathers, the harsh elements could impact more on the children. Another element to take into account is that, in general, boys experience more harshness than girls, especially from fathers, in the family and other socialization contexts [69,71]. Finally, because fathers use to be more peripheral in children and adolescent education, the presence of this factor could suggest a more generalized pattern of relational violence and maltreatment from the father within the family [29,69]. Differences in the effect of verbal harshness and warmth related to gender are nuanced by their cultural specific context. Social expectations about the maternal and paternal role will influence the subjective perception of the adolescent, aside from their unique characteristic [69,72].

These results support previous research that proposes harsh and warm parental practices as two independent dimensions with differentiated effects [69,73,74], even in the neurodevelopment of children [75,76]. The impact of an adverse family climate and harsh parental practices on emotion regulation, evidenced by the relationship between offensive communication and psychologic distress, is worth noticing. At the same time, their link with PASNT denotes failure in developing a secure attachment, with the consequent epistemic distrust that can favor victimization.

A limitation of this transversal study is the impossibility to confirm causality or the predictive value of the variables, which would require a longitudinal design. The sample does not include late adolescents, in whom the influence of parents could fade, while the influence of the relationships with pairs and community increases. Finally, self-report data could be completed and contrasted by the parent’s and teacher’s reports. Even with this limitation, it makes a valuable contribution to several dimensions:

1. In the theoretical field, it suggests that parent–adolescent communication could be central to explaining the link between family variables, school bullying, and other violent behavior in adolescence, such as cyberbullying [36], dating violence [45], and child-to-parent violence [27,77]. Through its consequences over the development of trust (PAIA) or distrust (PASNT) toward authority and social norms, over emotion regulation (psychological distress) and social skills (community implication). The combination of these effects brings a broader understanding of the interactions and possible co-evolution between individual, relational and social factors. It also brings the possibility to reach a more integrative model to comprehend their continuity and circularity in determining social problems such as interpersonal violence and psychopathology, or resilience, and health.

2. In the prevention of violence in general, through the promotion of communication for peace: Encouraging warmth and indulgent socialization practices in parents, teachers, and other adults in contact with adolescents will sustain the development of epistemic trust. Emotion socialization and the learning and practice of social skills will facilitate adolescent’s inclusion in school and community environments, preventing bullying victimization, and enhancing positives developments toward health and wellbeing [78].

Extra familiar adults are invaluable to increase resilience in young with the most disadvantaged developmental environment [62,79]. This highlights the importance of offering warmth and inclusive community spaces, attractive for adolescents, through sport, cultural or citizen activities, supervised by adults that can provide the experience of a consistent caring and open communication to strengthen the development of epistemic trust.

3. Concerning the interventions, these results emphasize the need to bring specialized support to the victims aimed to lessen their psychological distress, encourage their social skills practice and improve emotion regulation processes, to facilitate their social inclusion and increase their wellbeing.

4. Finally, in the light of our findings, promotion, prevention, and intervention programs should use differentiated strategies for adolescents and adults to address gender differences, since gender is a transversal variable that influences parenting and socialization processes through explicit and implicit ways [46] affecting perceptions, cognitions, and actions of everyone.

## 5. Conclusions

In conclusion, this evidence highlights the relevance of promoting warmth and sensitive parental and teaching practices and eradicating harsh socialization practices. Removing offensive communication will reduce their deleterious effects over adolescent emotion regulation and socialization ability, while increasing open communication will potentiate their psychosocial adjustment, protecting them from bullying victimization. The community is a valuable resource to adolescent social practice that can provide different life experiences than the experience in families and schools, with a high potential to promote relational skills and emotion socialization and enhance epistemic trust. Hence, it should be included in the plans and strategies aimed to prevent adolescent violence in general, bullying in particular, and their adverse effects on adolescent development, mental health, life and future.

## Figures and Tables

**Table 1:** Pearson correlations among variables, descriptive statistics, and *t* Student proof.

Variables in Study	1	2	3	4	5	6	7	8	9	10	11	12	13
1.RV	1												
2.DPV	0.72 **	1											
3.DVV	0.84 **	0.73 **	1										
4.PD	0.38 **	0.25 **	0.37 **	1									
5.OPCM	−0.18 **	−0.16 **	−0.18 **	−0.29 **	1								
6.OFCM	0.20 **	0.20 **	0.19 **	0.26 **	−0.14 **	1							
7.OPCF	−0.16 **	−0.11 **	−0.16 **	−0.29 **	0.66 **	−0.06 *	1						
8.OFCF	0.21 **	0.21 **	0.19 **	0.19 **	−0.04	0.70 **	−0.08 **	1					
9.COMI	−0.16 **	−0.11 **	−0.15 **	−0.22 **	0.28 **	−0.15 **	0.31 **	−0.13 **	1				
10.COP	−0.08 **	−0.03	−0.09 **	−0.231	0.25 **	−0.08 **	0.24 **	−0.04	0.46 **	1			
11.ISS	−0.12 **	−0.08 **	−0.13 **	−0.214	0.28 **	−0.16 **	0.29 **	−0.13 **	0.54 **	0.51*	1		
12.PAIA	−0.19 **	−0.18 **	−0.20 **	−0.229	0.31 **	−0.20 **	0.30 **	−0.21 **	0.24 **	0.22 *	0.24 **	1	
13.PASNT	0.13 **	0.18 **	0.13 **	0.164	−0.13 **	0.23 **	−0.09 **	0.20 **	−0.06 *	−0.08*	−0.05	−0.09 **	1
M/DT boys	1.7/0.8	1.6/0.7	1.8/0.8	2.1/0.8	3.6/1	1.9/1	3.4/1.1	1.9/1	2.9/0.6	2.6/0.6	2.7/0.4	2.7/0.7	1.8/0.7
M/DT girls	1.7/0.8	1.4/0.5	1.7/0.8	2.50/0.9	3.5/1.1	1.9/0.9	3.1/1.1	1.8/0.9	2.8/0.6	2.5/0.7	2.6/0.5	2.7/0.7	1.6/0.7
***t***	−0.027	7.38 ***	2.37 *	−8.73 ***	1.79	−1.16	0.84	3.79 ***	2.13 *	1.78	1.78	−0.56	4.07 ***

Note: * *p* < 0.05 (bilateral); ** *p* < 0.01 (bilateral); *** *p* < 0.001 (bilateral); RV: Relational Victimization; DPV: Direct Physical Victimization; DVV: Direct Verbal Victimization; PD: Psychological Distress; OPCM: Open Communication Mother; OFCM: Offensive Communication Mother; OCF: Open Communication Father; OFCF: Offensive Communication Father; COMI: Community Implication; COP: Community Participation ISS: Informal systems Social Support; PAIA: Positive Attitude toward Institutional Authority; PASNT: Positive Attitude toward Social Norms Transgression.

**Table 2:** Centroids of discriminant function for each group.

Group	Function
	1
1. No victimization	−0.098
2. High victimization	1.256

**Table 3:** Matrix structure.

Independent Variable	Value of Function 1 (Victimization)
Psychological distress	0.799
**OFCM**	0.526
OFCF	0.501
**PASNT**	0.400
Community implication	−0.361
**PAIA**	−0.340
OPCM	−0.333
**OPCF**	−0.166
Informal systems social support	−0.115
**Community participation**	−0.046

Note: OFCM: Offensive Communication with Mother; OFCF: Offensive communication with Father; PASNT: Positive Attitude toward Social Norms Transgression; PAIA: Positive Attitude toward Institutional Authority; OPCM: Open Communication with Mother; OPCF: Open Communication with Father.

**Table 4:** Results of classification of victimization groups.

No Victims and Highly Victimized			Predicted Membership Group		Total
			No Victims	High Victimization	
Original	Count	No victimization	821	259	1080
		High victimization	23	61	84
		Ungrouped cases	203	172	375
	Percentage	No victimization	76%	24%	100%
		High victimization	27.4%	72.6%	100%
		Ungrouped cases	54.1%	45.9%	100%

Note: a 75.8% of the original groups were classified correctly.

**Table 5:** Linear Regression Analyses.

Variables	B	Standard Error	Beta	*p*-Value	R^2^
Step 1					0.005
Gender—woman	−0.100	0.034	−0.074	0.004 **	
Age—15 to 16 years	−0.009	0.040	−0.006	0.815	
Step 2					0.178
Gender—woman	−0.182	0.096	−0.134	0.000 ***	
Age—15 to 16 years	−0.069	0.032	−0.044	0.060	
PD	0.289	0.037	0.359	0.000 ***	
PAIA	−0.118	0.020	−0.119	0.000 ***	
PASNT	0.071	0.023	0.072	0.002 **	
Step 3					0.195
Gender—woman	−0.179	0.032	−0.132	0.000 ***	
Age—15 to 16 years	−0.068	0.036	−0.043	0.063	
PD	0.263	0.020	0.326	0.000 ***	
PAIA	−0.083	0.025	−0.083	0.001 **	
PASNT	0.046	0.024	0.046	0.054	
OPCM	−0.038	0.016	−0.059	0.017 *	
OFCM	0.015	0.024	0.021	0.521	
OFCF	0.082	0.024	0.113	0.001**	
Step 4					0.197
Gender—woman	−0.182	0.032	−0.135	0.000 ***	
Age—15 to 16 years	−0.066	0.036	−0.041	0.072	
PD	0.259	0.021	0.322	0.000 ***	
PAIA	−0.077	0.025	−0.077	0.002 **	
PASNT	0.046	0.024	0.046	0.053	
OPCM	−0.033	0.016	−0.052	0.042 *	
OFCM	0.015	0.024	0.020	0.539	
OFCF	0.080	0.024	0.110	0.001 **	
COMI	−0.043	0.026	−0.040	0.101	

Note: * *p* < 0.05; ** *p* < 0.01; *** *p* < 0.001. PD: Psychological Distress; PAIA: Positive Attitude toward Institutional Authority; PASNT: Positive Attitude toward Social Norms Transgression; OPCM: Open Communication with Mother; OFCM: Offensive Communication with Mother; OPCF: Open Communication with Father; OFCF: Offensive communication with Father; COMI: Community Implication.
